# PDGFR in PDGF-BB/PDGFR Signaling Pathway Does Orchestrates Osteogenesis in a Temporal Manner

**DOI:** 10.34133/research.0086

**Published:** 2023-05-05

**Authors:** Fangqian Wang, Yuxiao Ye, Zengjie Zhang, Wangsiyuan Teng, Hangxiang Sun, Xupeng Chai, Xingzhi Zhou, Jiayu Chen, Haochen Mou, Yinwang Eloy, Xiaoqiang Jin, Liang Chen, Zhenxuan Shao, Yan Wu, Yue Shen, An Liu, Peng Lin, Jianwei Wang, Xiaohua Yu, Zhaoming Ye

**Affiliations:** ^1^ Department of Orthopedic Surgery, the Second Affiliated Hospital, Zhejiang University School of Medicine, Hangzhou City, Zhejiang Province, PR China.; ^2^ Orthopedics Research Institute of Zhejiang University, Hangzhou City, Zhejiang Province, PR China.; ^3^ Key Laboratory of Motor System Disease Research and Precision Therapy of Zhejiang Province, Hangzhou City, Zhejiang Province, PR China.; ^4^ School of Material Science and Engineering, University of New South Wales, Sydney 2052, Australia.

## Abstract

Platelet-derived growth factor-BB (PDGF-BB)/platelet-derived growth factor receptor-β (PDGFR-β) pathway is conventionally considered as an important pathway to promote osteogenesis; however, recent study suggested its role during osteogenesis to be controversial. Regarding the differential functions of this pathway during 3 stages of bone healing, we hypothesized that temporal inhibition of PDGF-BB/PDGFR-β pathway could shift the proliferation/differentiation balance of skeletal stem and progenitor cells, toward osteogenic lineage, which leads to improved bone regeneration. We first validated that inhibition of PDGFR-β at late stage of osteogenic induction effectively enhanced differentiation toward osteoblasts. This effect was also replicated in vivo by showing accelerated bone formation when block PDGFR-β pathway at late stage of critical bone defect healing mediated using biomaterials. Further, we found that such PDGFR-β inhibitor-initiated bone healing was also effective in the absence of scaffold implantation when administrated intraperitoneally. Mechanistically, timely inhibition of PDGFR-β blocked extracellular regulated protein kinase 1/2 pathway, which shift proliferation/differentiation balance of skeletal stem and progenitor cell to osteogenic lineage by upregulating osteogenesis-related products of Smad to induce osteogenesis. This study offered updated understanding of the use of PDGFR-β pathway and provides new insight routes of action and novel therapeutic methods in the field of bone repair.

## Introduction

Platelet-derived growth factor (PDGF) is one of the few recombinant growth factors for bone regeneration approved by Food and Drug Administration (FDA) in clinic due to its potent regulatory function during bone repairing. Structurally, PDGF family growth factor is composed of polypeptide dimers linked by disulfide bonds, which is usually released by platelet degranulation after blood vessel damages [1,2]. Conventionally, the primary role of PDGFs during bone healing is to act as chemokines and mitogens to stimulate recruitment and proliferation of osteoprogenitor cells such as mesenchymal stem cells (MSCs) [3], which in turn promote formation of new bone. PDGF-BB is the most popularly used PDGF family member due to its capability to activate multiple PDGF receptors and its distinguished ability in vasculogenesis [4]. Therefore, medical devices incorporating PDGF-BB have been developed by combining with biomaterials such as beta-tricalcium phosphate (β-TCP) to achieve local delivery and improve bone healing [5–7]. For instance, Augment developed by Wright Medical, which is composed of PDGF-BB and β-TCP, has been approved by FDA in treatment of minor bone defect or arthrodesis of facet joints [8–10] and furthermore considered the alternative to autogenous graft bone [8]. However, the therapeutic efficacy of PDGF-BB appeared to be unsatisfactory according to many researches. On one hand, even if PDGF-BB is combined with various auxiliary osteogenic materials, it still could not show positive effects in bone repair [11–13]. On the other hand, PDGF-BB also antagonized the effect of bone morphogenetic protein-2 (BMP-2) and interferes with the normal osteogenesis process [14]. Therefore, the role of PDGF-BB has been questionable in the field of bone healing.

Recently, PDGF-PDGF receptor-β (PDGFR-β) signaling pathway was identified as an important functional mediator for osteoprogenitor cell regulation during bone repairing. PDGF-BB and its receptor tyrosine kinas PDGFR-β are highly expressed during fracture healing [1,15], which demonstrated strong mitogenic, chemotactic, and angiogenic effects for bone healing [16]. For example, PDGF-BB/PDGFR-β signaling activation promoted skeletal stem and progenitor cells (SSPCs) [17] and MSC proliferation through Akt and extracellular regulated protein kinase (Erk) signaling [18]. PDGF-BB can also induce migration of smooth muscle cell and endothelial progenitor cells to promote angiogenesis and stabilize newly formed vasculature [19–21]. On the other hand, recent studies also reported that the role of PDGF pathway appeared to be very diverse during bone healing, which led to controversial results both in research and clinical data. Böhm et al. [17] reported that loss of PDGFR-β in Conditional Knock Out (cKO) mice resulted in undersized but densely mineralized callus, suggesting that inhibition of PDGF pathway could impair the proliferative responses of SSPCs while accelerating their osteogenic differentiation toward bone regeneration. Likewise, the research in periosteum-derived progenitor cells showed that PDGF-BB/PDGFR-β signaling exerts inhibitory effects on direct bone osteogenesis by activating ERK 1/2 signaling but downstream suppressing the canonical BMP-2/Smad pathway [22]. More particularly, PDGFR-β promotes SSPC survival and expansion at the early stage of bone healing via maintaining their immature, proliferative status while it impairs osteogenic differentiation of SSPC toward mature osteoblasts at the late stage of bone regeneration. These researches suggest that PDGF-PDGFR-β pathway plays a pivotal role in guiding the delicate balance of SSPC proliferation and differentiation, which might offer opportunities to develop novel therapeutic approach for bone healing. For other related osteocytes, studies had also demonstrated that PDGF-BB directly stimulated osteoclastic bone resorption effect [23].

Although PDGF-PDGFR-β signaling pathway has been conventionally harnessed as proregenerative cue to promote bone healing, ample evidence supported that inhibition of this pathway substantially enhanced differentiation of SSPC toward osteogenic lineage. Thus, we hypothesized that inhibition of PDGFR-β in a timely manner by systemically administration of PDGFR-β inhibitor would push SSPC differentiation toward mature osteoblast, which leads to accelerated bone regeneration.

Herein, we aimed to improve critical size bone defect healing by shifting the proliferation and differentiation balance of bone marrow-derived mesenchymal stem cells (BMSCs) toward osteogenic lineage by blocking PDGFR-β using its specific inhibitor SU-16f. We firstly screened a series of PDGF-BB/PDGFR-β inhibitor treatment conditions on human MSCs (hMSCs) to validate the pro-osteogenic role of PDGFR-β inhibitor. Then, we took this model further to optimize its impact on osteogenesis via timely control over PDGF-BB/PDGFR-β inhibitor combinations. The impact of PDGFR-β inhibitor on critical size bone defect healing was then evaluated in conjunction with calcium phosphate (CaP)/extracellular matrix (ECM) composite scaffolds implanted into the defect sites. Various administration routes were evaluated with timely control to emulate the in vitro results. Mechanistically, the temporal regulation in our study was related to Smad and Erk downstream of the PDGF-BB/PDGFR-β pathway. Early cell proliferation and vascular repair depend on Erk pathway expression, inhibiting the expression of downstream osteogenesis-related products of Smad, induced by PDGFR-β activation, which suggests that PDGF-BB has a relative inhibitory effect on osteogenic repair.

We found that inhibiting PDGF-BB/PDGFR-β pathway promotes osteogenic differentiation of MSCs and healing effect of large-area bone defects, which subverts the current clinical understanding of the use of PDGF-BB and provides new insight routes of action and novel therapeutic method in the field of bone repair.

## Results and Discussion

### Local release of PDGF-BB has no apparent healing effect in bone defects

**Fig. 1.**
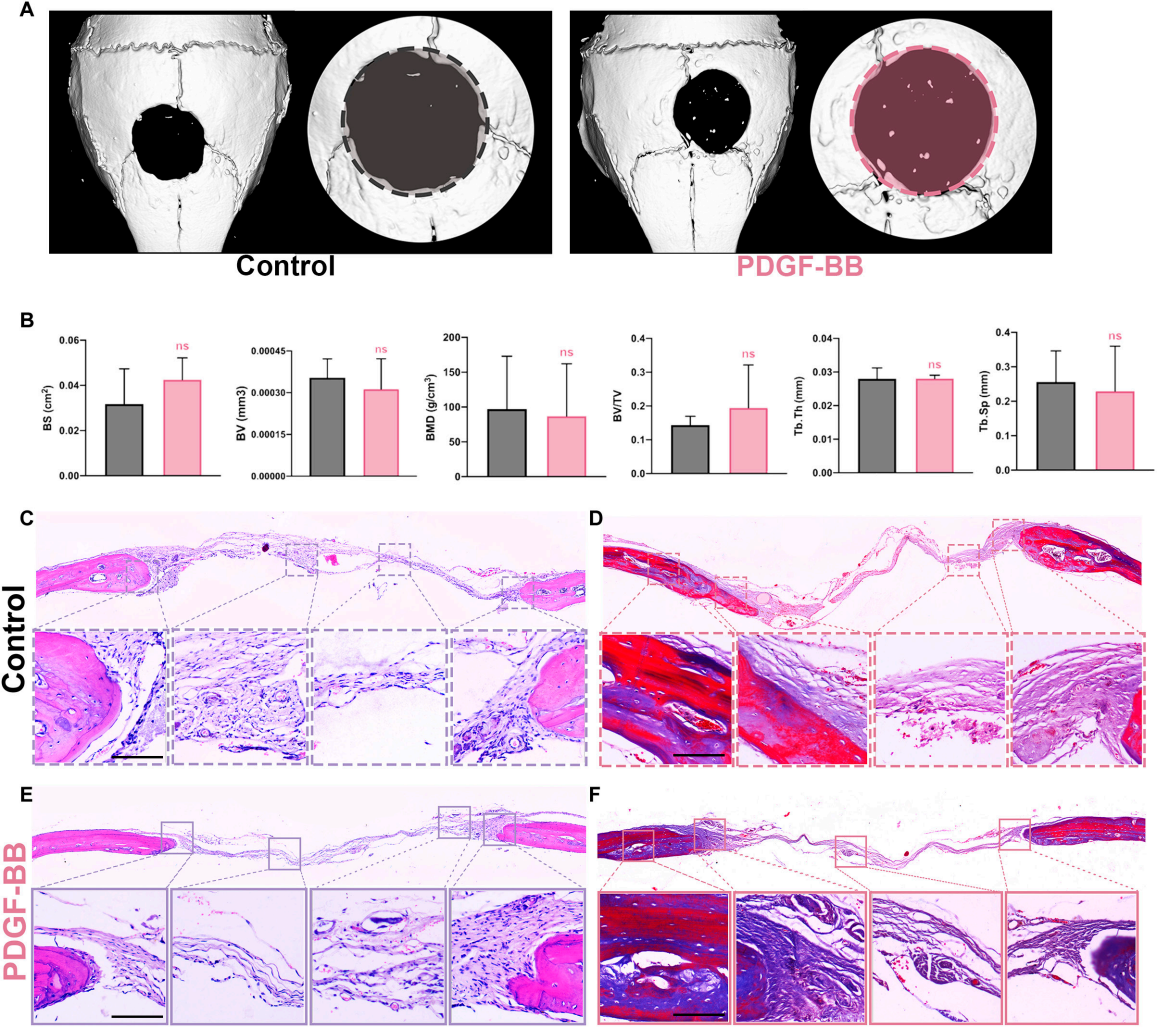
Local rapid release of PDGF-BB has no obvious osteogenesis effect on critical bone defect. (A) The reconstructions of representative calvarial models (average of the individual samples) measured by micro-CT. Original defect area is shaded with a dashed outline (control, black; PDGF-BB [1.0 ug], red). (B) The quantitative analysis of micro-CT reconstruction of CSD model (control, black; PDGF-BB, red). Data are means ± standard error of the mean (SEM). *n* = 5 or 6. Student *t* test. ns *P* ≥ 0.05. (C and E) The H&E staining image of each group (control, dotted line; PDGF-BB, full line). Scale bar, 50 μm. (D and F) The Masson staining of each group (control, dotted line; PDGF-BB, full line). Scale bar, 50 μm. BS, bone surface; BV, bone volume; BMD, bone mineral density; TV, tissue volume; Tb.Th, trabecular thickness; Tb.Sp, trabecular separation.

Controversial results have been reported regarding the healing effect of PDGF on bone defects [11–13]. We first constructed a PDGF-GelMa hydrogel delivery system to verify the effect of sustained PDGF on bone regeneration in cavalier defect models. GelMa formed a nonflowing solid (Fig. S1A) through optical cross-linking. By above steps, PDGF-GelMa hydrogel slices were prepared to fill the defect area of a mouse (Fig. S1B). Lyophilized GelMa hydrogel appeared porous under scanning electron microscopy (Fig. S1C). Further characterization showed that GelMa had good absorption and degradation properties. Figure S1D shows that freeze-dried GelMa absorbed water rapidly in a short period of time, reaching 8 times the weight in 100 min. PDGF-GelMa hydrogel degrades for 2 weeks (Fig. S1E). The release of PDGF-BB increased fast at first and then slowed down, reaching more than 80% in the first 2 weeks (Fig. S1F). Next, we made the GelMa hydrogel loaded with PDGF-BB into a suitable size and constructed a mouse skull critical bone defect model according to the surgical sequence (Fig. S2). After implanting the GelMa hydrogel for 2 months, micro–computed tomography (micro-CT) scan of the mouse defect skull area showed that control and PDGF-BB groups both had limited newly formed bone, indicating PDGF-BB did not have marked bone repair effect (Fig. 1A). Micro-CT quantification showed there was no significant difference between the 2 groups of specimens in terms of bone surface, bone volume, bone mineral density, and other indicators (Fig. 1B). From the pathological staining of the cross-section of mouse calvaria, the tissue reaction of the 2 groups were similar in terms of new bone formation. Between the 2 groups of bone fragments, only thin fibrous tissue is connected and it can be seen that the thickness of the fibrous tissue is similar.

The above results suggest that sustained release of PDGF-BB had no significant effect on critical size bone defect healing. However, some literature showed that PDGF-BB enhanced bone formation when combined with other bone grafting materials. Such inconsistence motivated us to further explore the diverse roles of PDGF/PDGFR-β signaling pathway.

### Inhibition of PDGFR-β signaling promotes osteogenesis but negatively regulates MSC proliferation

The role of PDGF-BB/PDGFR-β pathway on osteogenesis has been controversial. In order to explore the role of the PDGFR-β signaling in promoting osteogenic differentiation of hMSCs, we used either PDGF-BB or SU-16f, a PDGFR-β inhibitor to treat hMSCs during their osteogenic differentiation for various periods to evaluate their influence.

**Fig. 2.**
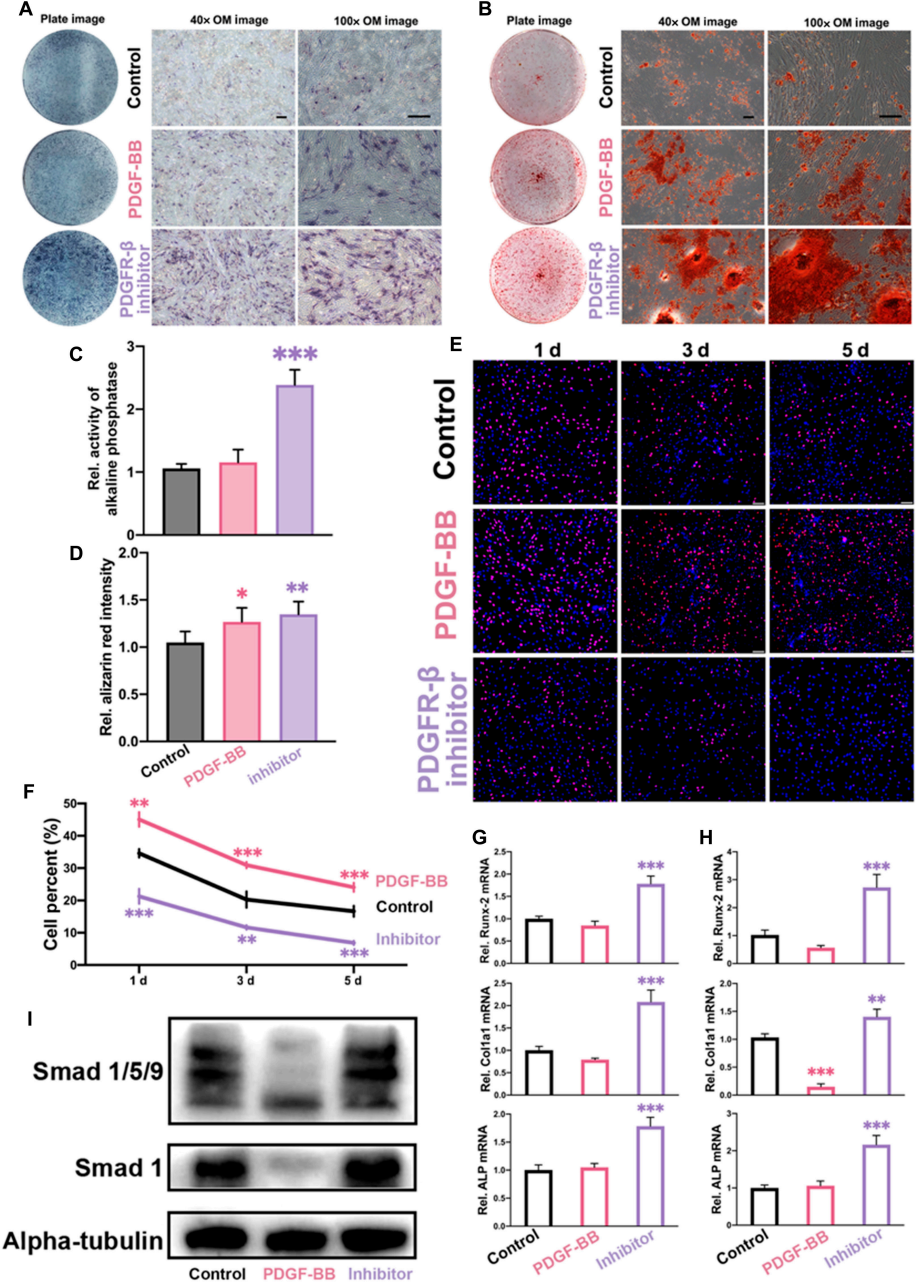
PDGF-BB activates the inhibitory effect of Erk 1/2 on the pathway of Smad-1/5/9 and promotes cell proliferation activity rather than osteogenic characterization while the PDGFR-β inhibitor (SU-16f) has the opposite effect. (A) ALP staining of the groups (control, black; 10 ng/ml PDGF-BB, red; 5 μM PDGFR-β inhibitor, purple) with low (40×) and high (100×) magnification of optical micrograph. Scale bar, 50 μm. (B) ARS of the groups (control, black; 10 ng/ml PDGF-BB, red; 5 μM PDGFR-β inhibitor, purple) with low (40×) and high (100×) magnification of optical micrograph. Scale bar, 50 μm. (C) Quantitative analysis of ALP staining of the groups. Data are means ± SEM. One-way ANOVA. ***P < 0.001. (D) Quantitative analysis of ARS of the groups. Data are means ± SEM. One-way ANOVA. **P < 0.01; ***P < 0.001. (E) EdU assay of the groups. (F) EdU assay of the groups in different time points (1, 3, and 5 d) with time trend and quantitative analysis. Data are means ± SEM. One-way ANOVA. **P < 0.01; ***P < 0.001. (G) PCR analysis of ALP, Col1a1, and Runx-2 mRNA of the groups with 5 d. Data are means ± SEM. One-way ANOVA. ns *P* ≥ 0.05; *P < 0.05; **P < 0.01; ***P < 0.001. (H) PCR analysis of ALP, Col1a1, and Runx-2 mRNA of the groups with 7 d. Data are means ± SEM. One-way ANOVA. ns *P* ≥ 0.05; *P < 0.05; **P < 0.01; ***P < 0.001. (I) WB band of Smad 1/5/9 and Smad 1 with 7 d.

Figure 2A and B represents the qualitative results of alkaline phosphatase (ALP) staining and alizarin red staining (ARS) of MSCs under different stimuli using a PDGF-BB or PDGFR-β inhibitor. Figure 2A and C shows a slight increase in the expression of ALP in MSCs after administration of PDGF-BB but without statistical differences. On the contrary, the expression of ALP was greatly increased with SU-16f achieving a nearly 2.5-fold increase. At the same time, the image from a high-power microscope showed that the distribution of ALP-positive cells effected by SU-16f was well proportioned, and the expression level of cells was much higher than that of the control group. Figure 2B and D shows that, under osteogenic differentiation condition, PDGF-BB caused the formation of calcium nodules in MSCs was slightly higher than that in the control group, which showed a slight increase in the number and density of calcium nodules. However, the number and density of calcium nodules increased significantly after administration of SU-16f, especially the formation of larger monolithic calcium nodules at the density level. The expression of ALP and the formation of calcium nodules are important indicators of the osteogenic performance of MSCs. The above results showed that the expression of these 2 markers is significantly higher administrated by SU-16f; on the contrary, PDGF-BB had no significant raise compared with control group.

Compared with the control group, neither PDGF-BB nor SU-16f was cytotoxic to MSCs (Figs. S3 and S4). PDGF-BB could even promote the increase of cell number through increasing the expression of 5-ethynyl-2'-deoxyuridine (EdU) in MSCs (Fig. 2E). On the contrary, SU-16f exhibited a clear inhibitory effect on hMSC proliferation. Quantitative data showed that the proportion of cells with proliferative activity of PDGF-BB group was about 110% of that with control group, while cultured with SU-16f was about 85% of that with control group (Fig. 2F). The potential of PDGF-BB for tissue healing shows the promotion of the proliferative capacity of MSCs, but the inhibition of SU-16f-mediated cell proliferation has more significant osteogenic characteristics. Mechanistically, SU-16f up-regulated the expression of osteogenesis-related genes ALP, Runx-2, and Col1a1 in MSCs at both 5 d (Fig. 2G) and 7 d (Fig. 2H). At the protein level, PDGF-BB/PDGFR-β signaling has 2-sided effects on osteogenesis for that PDGFR-BB could down-regulate the expression of Smad protein, the classic downstream target of BMP-2, and PDGFR-β inhibitor had no obvious up-regulation effect on it (Fig. 2I), which indicated that the therapeutic effect of clinical PDGF-BB use might be attributed to its stimulative effect on MSC proliferation.

Further, previous studies have shown that Erk 1/2, as a downstream signaling marker of PDGFR-β, can be activated after the targeting of PDGF-BB to the receptor [24,25]. Therefore, in order to further consolidate these findings at the cellular level, we selected Erk 1/2 inhibitor (U0126) to specifically inhibit Erk expression. Compared with the control group, ALP expression of MSCs increased after PDGFR-β inhibitor (SU-16f) and Erk inhibitor (Fig. S5A). Microscopic images showed no significant difference in cell density, but expression increased significantly after the use of both inhibitors. Furthermore, polymerase chain reaction (PCR) results showed that the expression of ALP, Runx-2, and Col1a1 was higher than that of the control group (Fig. S5B). However, the effect of PDGFR-β inhibitor was slightly stronger than that of the Erk group possibly because PDGFR-β was further upstream. Figure S5C showed the differences of Western blot (WB) bands at the protein level, showing that the expression of Smad 1 and Runx-2 increased with both inhibitors, but Osterix (Sp7) only increased under PDGFR-β inhibitor.

The role of MSCs is crucial for bone defect healing. PDGF-BB could promote the proliferation of MSCs, but PDGF-BB/PDGFR-β pathway had no positive role in promoting their osteogenic differentiation. Conversely, inhibition of this signaling pathway hindered cell proliferation but resulted in more pronounced osteogenic differentiation. Combined together, such PDGF-BB treatment might increase the number of osteoprogenitor cell at the early stage but may be detrimental to osteogenic differentiation of these cells. This might also explain the bone repairing effect of current clinical use of PDGF-BB when combined with osteoconductive materials as presence of PDGF-BB could promote proliferation of osteoprogenitor cells at the early stage of bone healing.

### Blockade of PDGFR-β pathway at the late stage under temporal control promotes osteogenesis in vitro and vivo

Our previous results suggest that PDGF-BB could promote the proliferation of MSCs while SU-16f could improve osteogenic differentiation of MSCs. In order to exploit their respective stimulative role, we next explored temporal regulation of PDGF-BB and PDGFR-β inhibitors during osteogenic differentiation. In a fixed culture cycle, we aimed to analyze the optimal effect of the PDGF-BB/PDGFR-β pathway in the osteogenic differentiation of MSCs by changing the duration of the action of PDGF-BB or SU-16f, respectively.

**Fig. 3.**
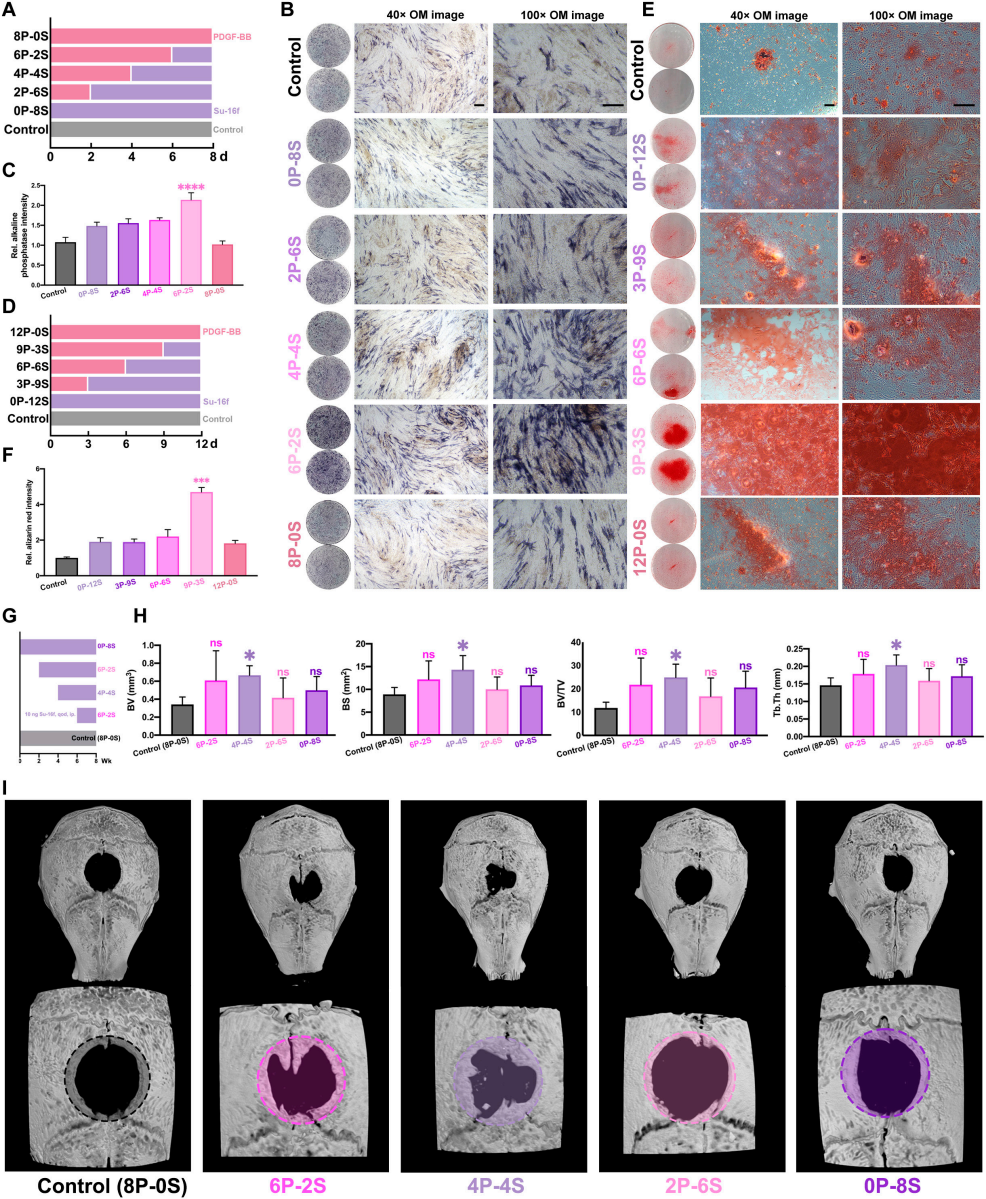
Blocked PDGFR-β in the late stage of osteogenesis can promote osteogenic function of hMSCs. (A) The schematic diagram of temporal control grouping with total time of 8 d (number before P or S respectively means cultured days of 10 ng/ml PDGF-BB or 5 μM SU-16f in the medium). (B) ALP staining with low (40×) and high (100×) magnification of optical micrograph. Scale bar, 50 μm. (C) Quantitative analysis of ALP analysis (control, black; 0P-8S, lilac; 2P-6S, modena; 4P-4S, amaranth; 6P-2S, light red; 8P-0S, red). Data are means ± SEM. *n* = 5 or 6. One-way ANOVA. ns *P* ≥ 0.05; **P < 0.01. (D) The schematic diagram of temporal control grouping with total time of 12 d (number before P or S respectively means cultured days of 10 ng/ml PDGF-BB or 5 μM SU-16f in the medium). (E) ARS with low (40×) and high (100×) magnification of optical micrograph. Scale bar, 50 μm. (F) Quantitative analysis of ARS (control, black; 0P-12S, lilac; 3P-9S, modena; 6P-6S, amaranth; 9P-3S, light red; 12P-0S, red). Data are means ± SEM. *n* = 5 or 6. One-way ANOVA. ns *P* ≥ 0.05; **P < 0.01. (G) The schematic diagram of vivo experiment grouping (number before P or S respectively means administration weeks of nontreatment or SU-16f). (H) The quantitative analysis of micro-CT reconstruction (control [0P-8S], black, given PBS by intraperitoneal injection [ip.] once every 2 d [qod.] after skull operation; 6P-2S, amaranth, given 10 ng SU-16f by ip. qod. in the 7th and 8th weeks after skull operation; 4P-4S, lilac, given 10 ng SU-16f by ip. qod. from 5th to 8th week after skull operation; 2P-6S, light red, given 10 ng SU-16f by ip. qod. from 3rd to 8th week after skull operation; 0P-8S, modena, given 10 ng SU-16f by ip. qod. after skull operation). Data are means ± SEM. *n* = 5 or 6. One-way ANOVA. ns *P* ≥ 0.05; **P < 0.01. (I) The reconstructions of representative calvarial models (average of the individual samples) measured by micro-CT. Original defect area is shaded with a dashed outline (control [0P-8S], black dotted line; 6P-2S, amaranth dotted line; 4P-4S, lilac dotted line; 2P-6S, light red dotted line; 0P-8S, modena dotted line).

PDGF and its receptor were detected after tissue injury takes place, especially in vascular tissue [26,27]. Therefore, we need to add physiological doses of PDGF-BB to simulate the release of PDGF-BB in vivo injury. According to the groupings indicated in Fig. 3A and D, when the total culture time was 8 or 12 d, the numbers before “P” indicated the time when PDGF-BB was added and the numbers before “S” indicated the time when SU-16f was added. Compared with the control group, ALP staining and quantitative analysis of 8 d showed no significant difference in expression with only PDGF-BB (8P-0S) and higher expression with only SU-16f (0P-8S) (Fig. 3B and C), which was similar to the results shown in previous Fig. 2. Interestingly, in groups with combination of PDGF-BB first and then SU-16f, the expression of ALP in cells was further improved, and ALP-positive cells in the same field of view increased in number and density. Moreover, from the perspective of the change trend, with the prolonged treatment of PDGF-BB, the highest expression of ALP could be achieved only when SU-16f (6P-2S) is administered in the late stage. The number of ALP-positive cells was the largest and the density is the highest, and the quantitative data also showed the highest expression levels. Correspondingly, Fig. 3E and F shows the results of AR staining: the difference between the single-dose groups is similar to the previous study, and the combined treatment resulted in more calcium nodules when PDGF-BB was given in the early stage and the late stage. After giving SU-16f, the number of calcium nodules reached the maximum with a large aggregation phenomenon from the integration of small mineralized particles to large calcium plaque. Temporal regulation study of PDGF-BB inhibitor showed that the highest expression of ALP, Runx-2, and Col1a1 was observed when SU-16f (6P-2S) was given only in the late stage (Fig. S6A). Gene expression in the short-term 4 d (Fig. S6B) showed that group with only SU-16f administered in the late period achieved the highest expression (Fig. S6C).

We further extended the concept of time regulation into the vivo study. As shown in Fig. 3G, mouse skull critical size defect (CSD) was created and allowed to heal for 8 weeks. These defects were divided into 5 groups according to the 2-week division (“P” represented no addition of PDGFR-β inhibitor [self-secreted PDGF-BB in the body], and “S” stand for addition of SU-16f to inhibit PDGF-BB/PDGFR-β pathway). SU-16f was injected intraperitoneally at 10 ng per animal and administered every other day. Figure 3I shows the healing of skull defects in each group of mice under micro-CT, and quantitative data were quantitatively analyzed in Fig. 3H. Compared with the control group, there was no significant difference in tissue healing between the mice treated with SU-16f for whole 8 weeks (0P-8S) and the last 6 weeks (2P-6S). The circular bone defect areas were round with blunt edges, but the overall defect areas were not repaired. The bone mass and bone area increased to a certain extent of the mice treated with SU-16f from the 3rd week for 6 weeks (6P-2S), but there was no statistical difference compared with the control group due to intergroup differences. The bone mass and bone area of the mice treated with SU-16f from the 5th week for 6 weeks (4P-4S) increased. The edge of the circular bone defect showed irregular healing, and the bone defect areas of 4P-4S group were decreased.

By combining PDGF-BB and PDGFR-β inhibitor treatment in a temporal manner, we showed that optimal osteogenic differentiation of hMSC could be achieved in vitro. These data clearly demonstrated the differential role of these 2 agents during hMSC culture: PDGF-BB exerts a stimulative role during hMSC proliferation while PDGFR-β inhibitor significantly pushes hMSC down to the osteogenic lineage. Neither PDGF-BB or PDGFR-β inhibitor alone could achieve the best osteogenic effect, and only the administration of PDGF-BB in the early stage and SU-16f in the later stage could achieve the maximum osteogenic performance. The results of ALP and ARS staining corresponded to the results of ALP-positive MSCs in the later stage of osteogenic differentiation to form osteoblasts and mineralization to precipitate calcium. The formation of dense ALP-positive cells and lamellar mineralized nodules shown in the osteogenic characterization experiments verified that blockage of PDGF/PDGFR-β pathway via SU-16f could enhance osteogenic differentiation of MSCs, which might be leveraged to improve bone regeneration in the context of SSPC-mediated bone repairing. At the same time, the in vitro results were also been verified in vivo. The qualitative and quantitative data of micro-CT on bone repair showed that the block of PDGF-BB/PDGFR-β pathway with SU-16f intervention at the late stage could promote bone defect repair. This might be due to the secretion of PDGF-BB after bone defect, which promotes the proliferation of MSCs and vascular endothelial cells [28], as well as the reconstruction of vascularization network [29], all of which provided the prior conditions for promoting osteogenic differentiation and mineralization after pathway inhibition. PDGF-BB alone or its inhibitor action did not achieve such a repair effect. Besides, we also found that the in vivo and in vitro time nodes do not coincide perhaps because of a greater abundance of cytokines in vivo and more tissue healing pathways [30–33].

### Late blockade of the PDGFR-β pathway promotes the healing of calvarial bone defects in mice with ECM scaffolds

**Fig. 4.**
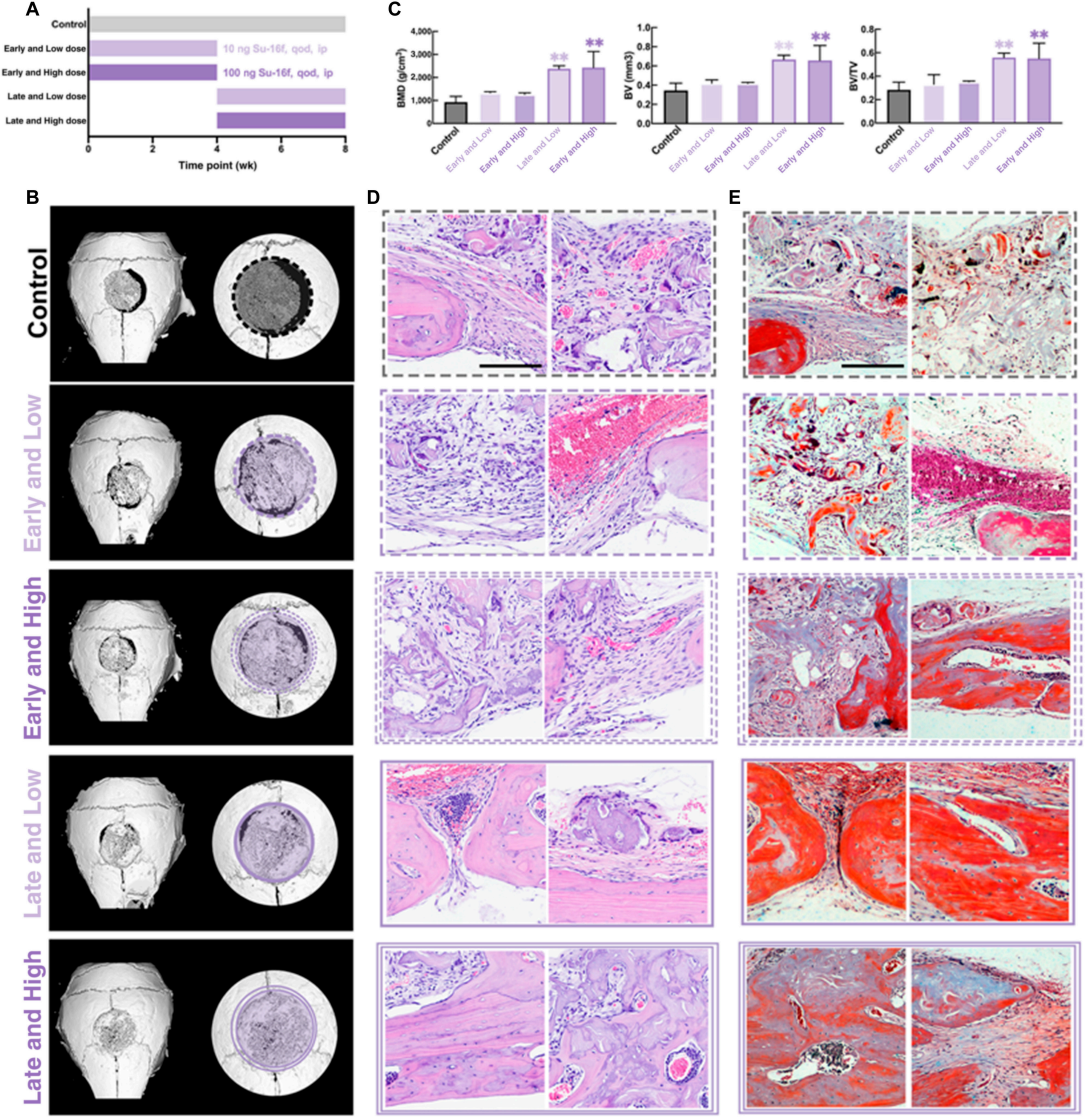
The administration of PDGFR-β inhibitor (SU-16f) in the late stage of osteogenesis of bone CSD mouse can significantly promote bone healing and mineralization with assistance of ECM scaffold. (A) The drug delivery schematic diagram of control and PDGFR-β inhibitor serious groups with dose control (Low dose, 10 ng SU-16f qod. ip.; High dose, 100 ng SU-16f qod. ip.) and temporal control (Early, the 1st to 4th week after skull CSD operation; Late, the 5th to 8th week after skull CSD operation). (B) The reconstructions of representative calvarial models (average of the individual samples) measured by micro-CT. Original defect area is shaded with a dashed outline (control, black dotted line, given PBS by ip. qod. after skull operation; Early/Low, purple single dotted line, given 10 ng SU-16f, ip. qod. from the 1st to 4th week; Early/High, purple double dotted line, given 100 ng SU-16f, ip. qod. from the 1st to 4th week; Late/Low, purple single full line, given 10 ng SU-16f ip. qod. from the 5th to 8th week; Late/High, purple double full line, given 100 ng SU-16f ip. qod. from the 5th to 8th week). (C) The quantitative analysis of micro-CT reconstruction (control, black; Early/Low, lilac without board; Early/High, modena without board; Late/Low, lilac with board; Late/High, modena with board line). Data are means ± SEM. *n* = 5 or 6. One-way ANOVA. ns *P* ≥ 0.05; **P < 0.01. (D) The H&E staining image of each group (control, black dotted line; Early/Low, purple single dotted line; Early/High, purple double dotted line; Late/Low, purple single full line; Late/High, purple double full line). Scale bar, 50 μm. (E) The Masson staining of each group (control, black dotted line; Early/Low, purple single dotted line; Early/High, purple double dotted line; Late/Low, purple single full line; Late/High, purple double full line). Scale bar, 50 μm.

On the basis of our finding from in vitro studies, we next evaluated the effect of PDGFR-β inhibitor on bone healing with a mouse calvarial defect model (Fig. S7). To assist the osteogenesis process in the bone defects, a CaP/ECM composite scaffold was used to be implanted into the defect sites, harnessing its osteoconductive properties, as well as its scaffolding function [34–36]. The grouping of vivo experiments was shown in Fig. 4A that, in addition to the control group, we also designed time gradients of early (first 4 wk after operation) and late (the 5th wk to the 8th week) dosing as well as concentration gradients of high and low concentrations. It is worth noting that most of the current osteogenic therapeutic drugs rely on the delivery system, which increases the complexity of the treatment, but the small molecule SU-16f has excellent efficacy after intraperitoneal injection.

The influence of SU-16f treatment on bone defect healing was clearly shown in Fig. 4B. From micro-CT scanning, we observed the defects treated with Late and High condition were filled with more new bone. Quantitative measurement of micro-CT found that compared to the 2 groups injected intraperitoneally in the early stage (Early and Low as well as Early and High), the mice injected in the late stage (Late and Low as well as Late and High) formed more new bone and mineral volume (Fig. 4C). Specifically, a larger mineralized area was formed on the edge of the scaffold, and the gap of the scaffold was more seamlessly filled with new bone in the late administration groups. Also, quantitative data showed that the bone density and bone mass in the defect area of ​​the late administration group were significantly higher. It is noteworthy that the repair effect of SU-16f on bone defects did not seem to be dose-dependent. The bone regeneration at defect sites were further evaluated by pathological staining (Fig. 4D and E). Tissue reaction in early groups (offered administration in the first 4 wk) showed no difference compared to the control group as only fibrous tissue was observed under hematoxylin and eosin (H&E) staining and Masson staining. In contrast, large bone area filled with osteocytes were seen in the late groups (administrated from wk 5 to wk 8) with no hindrance on the cellular ingrowth and blood vessels formation, clearly indicating ingrowth of new bone under late administration of SU-16f.

The bone defect healing model clearly showed that late-stage administration of Su-16, a PDGFR-β inhibitor, significantly promoted new bone formation with the assistance of CaP/ECM scaffold. As shown in our results, early administration of SU-16f did not seem to have a significant effect on bone defect healing; however, late administration of this agent clearly enhanced bone healing by showing much more new bone. This might be attributed to the possibility that late-stage inhibition of PDGFR-β could significantly promote the differentiation of SSPC, which leads to more bone formation. On the contrary, prematurely intervening the PDGF-BB/PDGFR-β pathway to inhibit the effect of PDGF-BB would hinder the process of vascularization repair or MSC recruitment and proliferation, which might resulted in poor bone healing outcomes. Moreover, SU-16f, as a small-molecule drug with a molecular weight of only 386.44, could be conveniently administered intraperitoneally, while other osteogenic factors such as BMP-2 need to be delivered locally using a carrier material. At the same time, the high bioavailability provided by the small molecular structure also ensured that the PDGFR-β inhibitor also had biological effects at low administration concentrations. For this study, while providing a new treatment idea for the clinic, the convenient administration method was also beneficial to the further clinical transformation and the development of drug delivery system in the future. In particular, bone defect healing strategies mostly focus on growth factor-based therapeutics, which are vulnerable to various factors in vivo. Our study demonstrate a practical approach to achieve large-area bone defect repair effects by simple injection, which can be conveniently implemented and reduce patient burden. Furthermore, this approach is expected to offer a more precise and convenient treatment by cooperating with conventional bone graft substitutes.

### Late administration of a PDGFR-β inhibitor achieves material-independent bone defect repair

Although we demonstrated that systemic administration of SU-16f at the late stage of bone healing effectively improved bone regeneration when CaP/ECM scaffolds were implanted in the bone defects as bone grafts, the proregenerative effect of Su-16 in a scaffold-free defect healing model was not evaluated. Because of the clinical complexity and convenience of direct injection in treating bone defects and other related pathological scenario, we next attempted to treat bone defects using SU-16f without scaffolding materials. Therefore, we designed an in vivo model without adding scaffold material but injecting SU-16f intraperitoneally in the late stage to observe bone defect repair.

**Fig. 5.**
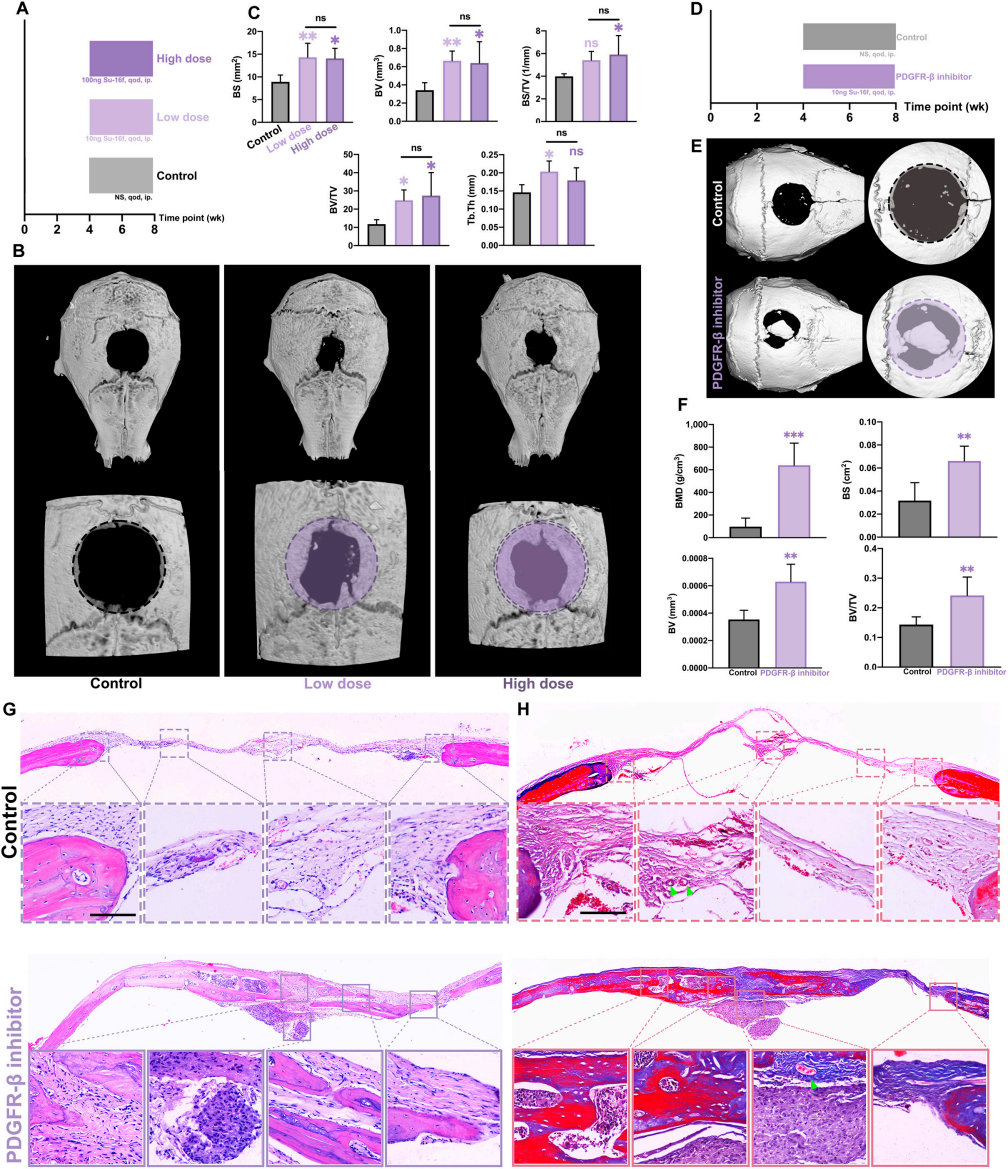
PDGFR-β inhibitor (SU-16f) which does not rely on biological materials and high dosage can also effectively promote bone healing in mice. (A) The drug delivery schematic diagram of control and PDGFR-β inhibitor with high dose (100 ng SU-16f, qod., ip.) and low dose (10 ng SU-16f, qod., ip.). (B) The reconstructions of representative calvarial models (average of the individual samples) measured by micro-CT. Original defect area is shaded with a dashed outline (control, black single dotted line; Low dose, purple single dotted line; High dose, purple double dotted line). (C) The quantitative analysis of micro-CT reconstruction of CSD model. Data are means ± SEM. *n* = 5 or 6. One-way ANOVA. ns *P* ≥ 0.05; **P < 0.01. (D) The drug delivery schematic diagram of control and PDGFR-β inhibitor (10 ng SU-16f, qod., ip.). (E) The reconstructions of representative calvarial models (average of the individual samples) measured by micro-CT. Original defect area is shaded with a dashed outline (control, black; PDGFR-β inhibitor, purple). (F) The quantitative analysis of micro-CT reconstruction of CSD model. Data are means ± SEM. *n* = 5 or 6. Student *t* test. ns *P* ≥ 0.05; *P < 0.05; **P < 0.01; ***P < 0.001. (G) The H&E staining image of each group (control, purple dotted line; PDGFR-β inhibitor, purple full line). Scale bar, 50 μm. (H) The Masson staining of each group (control, red dotted line; PDGFR-β inhibitor, red full line). Green triangle arrow, small blood vessels. Scale bar, 50 μm.

As shown in Fig. 5A, we set up 2 concentration gradients as previously designed, both of which were administered from the 5th week after the bone defect healing started. The high-concentration group and low-concentration group were given 100-ng SU-16f and 10-ng SU-16f each time, respectively, and the corresponding control group was given phosphate buffered saline (PBS). The mice in the 3 groups were given intraperitoneal injection of the drug every other day. Figure 5B and C shows the healing data of critical bone defect of mice calvarial without biomaterials under the action of high and low doses of SU-16f. Compared with the control group, both high- and low-concentration groups achieved bone defect healing effect through late administration (5th wk to 8th wk). It could be seen that the defect areas were reduced and irregular healing areas appear at the defect edges. There was no statistical difference in quantitative data such as bone mass or bone area between the 2 experimental groups with SU-16f. The in vivo results of concentration gradient groups were consistent with previous studies of ECM scaffold implantation.

As shown in Fig. 5D, the 2 groups of mice started to be intervened at the 5th week after creating calvarial defect. The control group was intraperitoneally injected with normal saline and the experimental group was intraperitoneally injected with low-concentration (10 ng per mouse) SU-16f according to above findings. As shown in Fig. 5E, the bone defect in the control group showed no detectable new bone formation, and the defect area remained its original size while a large portion of the bone defect in treatment group was covered by new bone, distributing at both periphery and center of the defect. Further, the quantitative results of defect repair by micro-CT, as shown in Fig. 5F, showed that the bone mineral density, bone area, bone mass, and other indicators of the experimental group were significantly improved compared to those of the control group. On the pathological level, H&E staining and Masson staining results (Fig. 5G and H) showed that there was no bone formation but thin fibrous tissue covered in defect area in the control group, which validated the modeling requirements for critical bone defects. The defect in the experimental group was repaired by a large amount of mineralized tissue accompanied by ingrowth of small blood vessels.

Although mouse calvarial defect model was employed as a proof-of-concept animal model here, it has been regarded as a high clinical relevant model. Data from the US Health Cost & Utilization Project showed that 12,700 craniotomies and craniectomies were performed in 2001. The national cost of the USA for this procedure was estimated to be approximately $549 million [37]. Therefore, the study on craniofacial fracture defect healing has received great attention and the calvarial CSD model in mice can be used to simulate this disease phenomenon [38]. At present, craniofacial bone defects still mainly rely on surgical procedures combined with autologous, allogeneic, or prosthetic materials [39,40]. Autograft or allograft materials are clinically limited by donor origin and associated harvest morbidity. The effectiveness of prosthesis materials is limited by unpredictable graft absorption, infection, structural failure, and unsatisfactory aesthetic results [39,40]. However, in recent years, the use of cytokines, including PDGF-BB, to treat skull defects and fracture healing is being investigated [41].

PDGF-BB is also partially bound to PDGFR-ɑ in vivo [42]. We further explored the role of PDGF-BB on the downstream pathway with different receptors after secretion in blood in Fig. S9. Preliminary results confirm that inhibition of PDGFR-β could promote bone defect healing. Both PDGFR-ɑ and PDGFR-β receptors were blocked by corresponding inhibitors (PDGFR-ɑ inhibitor AP24534 and PDGFR-β inhibitor SU-16f) from the 5th week to the 8th week (Fig. S9A). Figure S9B and C shows qualitative and quantitative interpretation of vivo data by micro-CT. Compared with the control group, the skull defect of mice treated with PDGFR-ɑ inhibitor did not heal significantly as the defect area did not decrease, which showed no statistical difference in bone mass and bone area. The above experiments might be because PDGF-BB was more specific bound to PDGFR-β in the bone defect area, so that the bone repair effect similar to PDGFR-β was not achieved after the late block of PDGFR-ɑ.

**Fig. 6.**
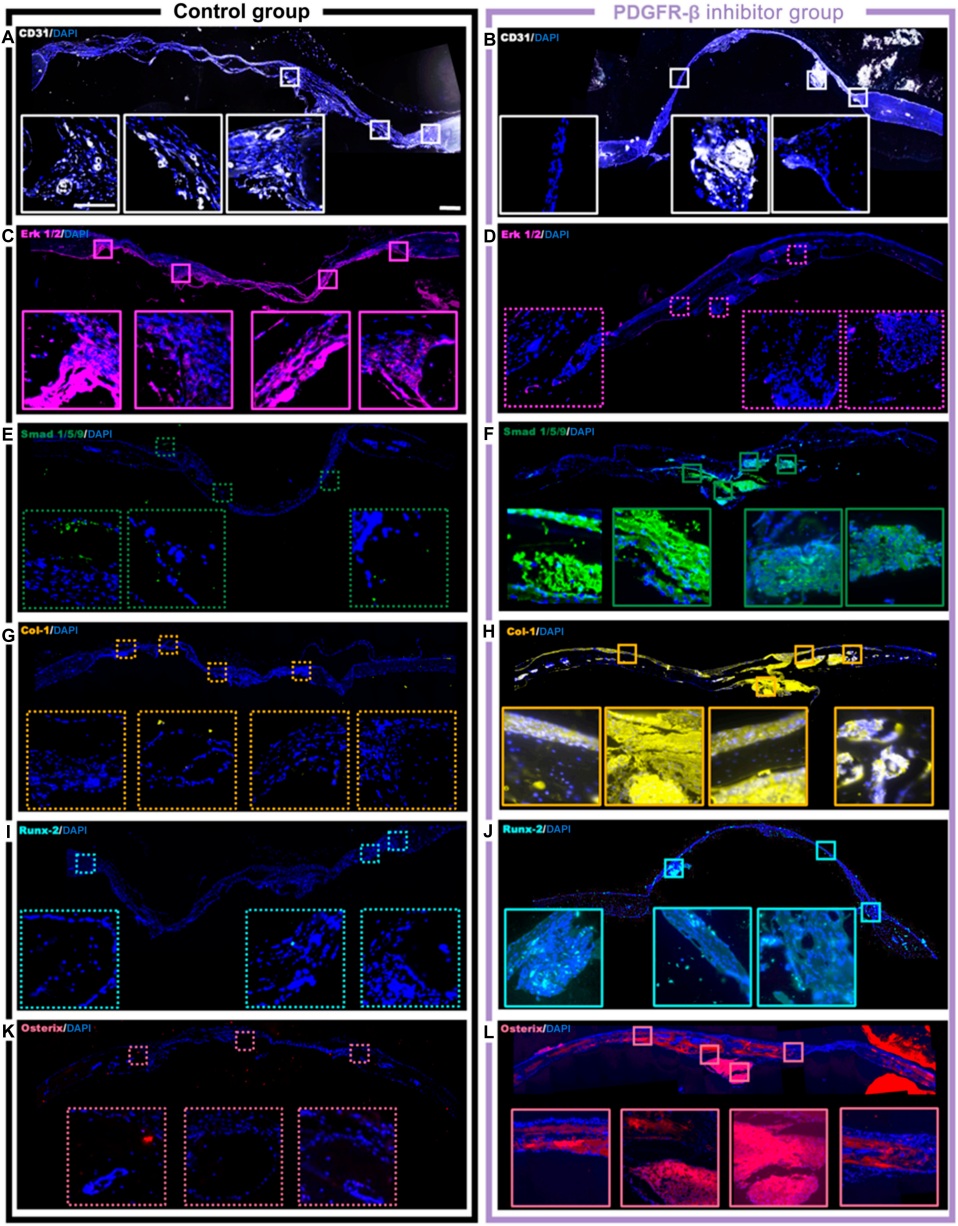
Immunofluorescence shows that the time-controlled delivery of PDGFR-β inhibitor (SU-16f) does not affect formation of blood vessels in the bone defect but increases osteogenic targets. (A and B) The fluorescence images with CD31 (white) and Dapi (blue) (2 groups with both white full line). Scale bar, 100 μm. (C and D) The fluorescence images with Erk 1/2 (amaranth) and Dapi (blue) (control, full line’PDGFR-β inhibitor, dotted line). (E and F) The fluorescence images with Smad 1/5/9 (green) and Dapi (blue) (control, dotted line; PDGFR-β inhibitor, full line). (G and H) The fluorescence images with Col1a1 (yellow) and Dapi (blue) (control, dotted line; PDGFR-β inhibitor, full line). (I and J) The fluorescence images with Runx-2 (cyan) and Dapi (blue) (control, dotted line, PDGFR-β inhibitor, full line). (K and L) The fluorescence images with Osterix (red) and Dapi (blue) (control, dotted line; PDGFR-β inhibitor, full line).

To further correlate PDGF-BB/PDGFR-β pathway with bone healing process, we performed immunofluorescence staining for markers associated with bone formation. We firstly stained for vascular marker CD31 as PDGF-BB can promote the proliferation of vascular endothelial cells, which enhance vascularization for tissue healing. There was no significant difference in the expression of angiogenic factor CD31 between the control group and the experimental group served with SU-16f (Fig. 6A and B). Secondly, immunofluorescent staining was performed for Erk 1/2 downstream of the canonical pathway PDGFR-β (Fig. 6C and D) and Smad 1/5/9 downstream of BMPR (Fig. 6E and F) to observe the level of osteogenesis-related protein expression. Blockade of PDGF-BB/PDGFR-β pathway led to significantly lower expression of Erk 1/2 than the control group but substantially upregulated Smad expression in the experimental group. Finally, we verified the expression of osteogenesis-related genes in the calvaria of mice in the control and experimental groups, including Cola1a (Fig. 6G and H), Runx-2 (Fig. 6I and J), and Osterix (Fig. 6K and L). Figure S10 shows quantitative data of the above markers. The expression of osteogenesis-related genes on the new bone in SU-16f group was significantly higher than that in the control group. Especially Osterix (Sp7) as a late osteogenic factor downstream of Runx-2, its significantly high expression suggested that late inhibition of the PDGF-BB/PDGFR-β pathway could significantly improve bone repair performance. These findings were also consistent with previous in vitro experiments. In summary, we have demonstrated that activation of the PDGF-BB/PDGFR-β pathway in the early stage and blocking it in the late stage achieved the best osteogenic healing effect.

**Fig. 7.**
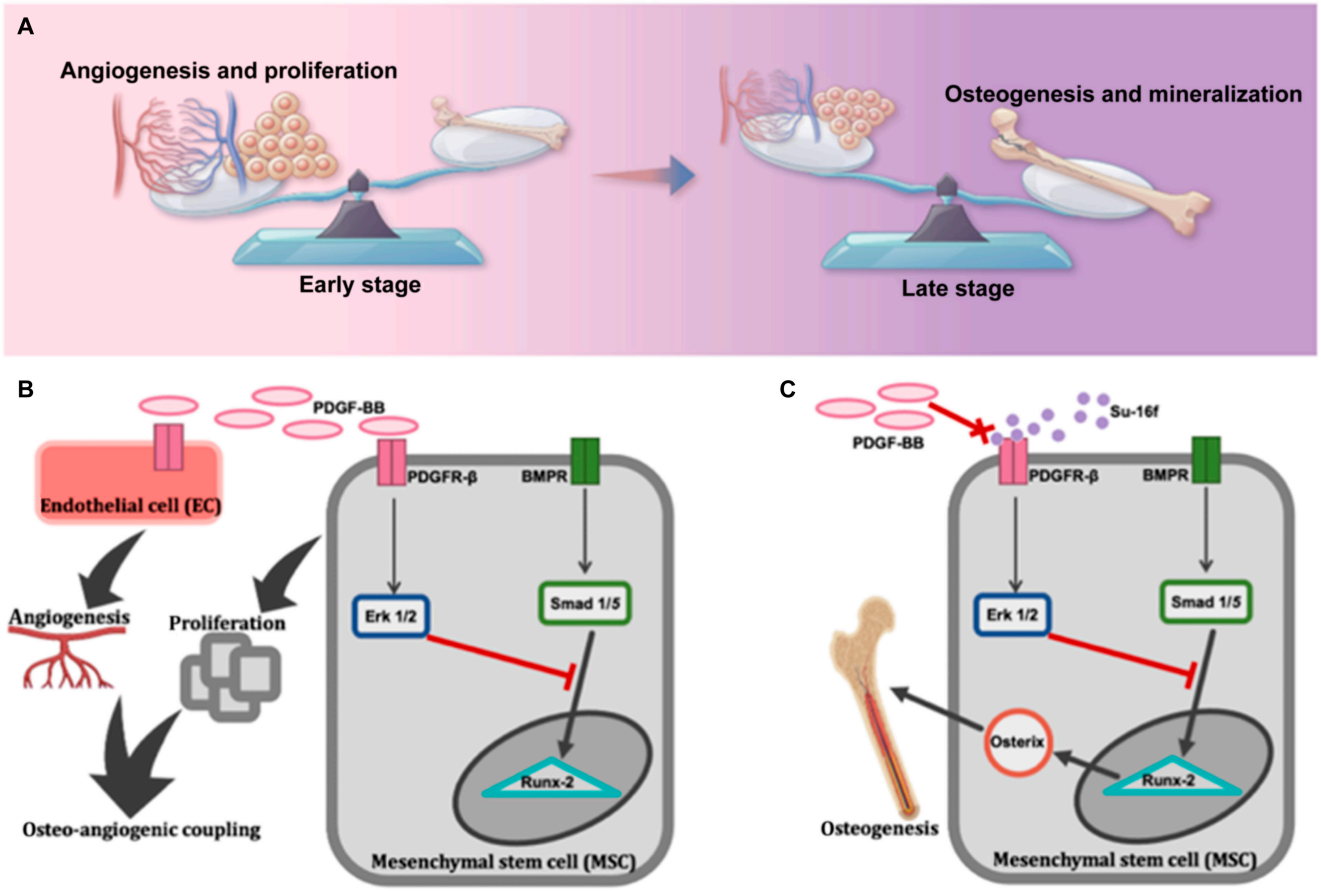
The abstract figure of the effect of PDGFR-β inhibitor (SU-16f) on pathways in late stage of bone healing. (A) The early stage of osteogenic repair focuses on cell expansion and angiogenesis, while the later stage should emphasize osteogenic differentiation and mineralization. (B) The abstract figure shows the role of PDGF-BB in bone repair in general. (C) The abstract figure shows SU-16f (PDGFR-β inhibitor) antagonizes PDGFR-β to promote bone repair.

Regarding the overarching strategy of achieving optimal bone healing, early-stage cell proliferation and late-stage mineralization need to be perfectly balanced. On one side, platelet degranulation releases PDGF-BB to promote cell proliferation and angiogenesis in the early stage in response to bone injury. On the other hand, inhibition of PDGF-BB/PDGFR-β pathway upregulates BMPR-Smad pathway, which is crucial for osteogenesis. On the basis of our findings, we propose a mechanism to specifically illustrate this balance-shifting process: as bone injury takes place, PDGF-BB is released by platelets and macrophages at early stage of bone healing. As it binds to PDGFR-β, Erk 1/2 is activated, which further promotes SSPC proliferation and inhibits their apoptosis; meanwhile, PDGF-BB downstream signaling reduces the express of canonical BMP-2/Smad pathway and its related gene expression, which substantially suppresses osteogenic differentiation of SSPC. In response, we chose to block PDGFR-β with SU-16f at the late stage of bone healing, which effectively shifts the proliferation/differentiation balance to the differentiation side via inhibition of Erk 1/2 pathway. In summary, we specifically blocked the PDGFR-β receptor by the small-molecule drug SU-16f in the late stage, so that effect of osteogenic healing can be achieved without affecting PDGF-BB effect in early stage. Rational use of this finding to inhibit the PDGF-BB/PDGFR-β pathway in the late stage of osteogenic healing not only ensured the proliferation of tissue cells but also promoted the process of osteogenic differentiation on a larger cell base to achieve optimal bone healing effect (Fig. 7).

At present, it is much accounted of biological materials in tissue repairing and many of them are FDA-approved for clinical conversion like 3D-print polycaprolactone scaffold [43], such as Osteoplug^TM^ proven to be safe over a 10-year period for craniotomy burr hole reconstruction [44] and reducing scalp-depression-related cosmetic handicaps in patients [45]. However, in orthopedics, bone repair cannot be achieved with biological scaffolds alone, which often need to be combined with other related drugs or biological components to achieve therapeutic effects, like β-TCP [46]. In contrast, PDGFR-β inhibitor can promote bone defect healing by injection, providing convenience for clinical use and carrying a new nonsurgical therapeutic idea for clinical practice. At the same time, PDGFR-β inhibitor, as a biological product, can be fully combined with biomaterials like 3D-print scaffold to achieve accurate time delivery and enhance clinical repair effect.

## Conclusion

In conclusion, here, we explored the possibility of using a PDGFR-β inhibitor, SU-16f, to improve critical size bone defect healing and clearly demonstrated that inhibition of PDGF-BB/PDGFR-β pathway using this molecule at the late stage of bone healing accelerated bone regeneration. Although such findings are somehow controversial with previous literature and clinical practice, our data supported that the role of PDGF-BB/PDGFR-β pathway at different stages of bone healing might vary dramatically. Specifically, fine tune of temporal administration of PDGFR-β inhibitor could exert a positive impact on osteogenesis. Thus, the findings from this study could be harnessed to combine with other therapeutics such as BMP-2, PDGF-BB, and bone graft substitutes, to further enhance their bone repairing performance at the late stage of bone healing. Our finding could put forward new requirements of PDGF-BB usage and also provided new ideas for the clinical treatment of large-area bone defects.

## Materials and Methods

### Preparation and characterization of GelMa hydrogel and ECM scaffold

GelMa hydrogel was purchased from Engineering For Life (Suzhou, Jiangsu, China). We used GelMa photo-cross-linking properties to shape the GelMa into a cylinder with a diameter of 3.5 mm and a height of 1 mm, which was the sample of the GelMa in this article. It was roughly characterized in a 2-ml glass bottle before and after ultraviolet cross-linking. In addition, for the hydrogel sheet containing PDGF-BB, 1.0 μg ​​of PDGF-BB was injected into each sheet before cross-linking and then mixed and cross-linked to form.

Scanning electron microscopy characterization: Surface morphology of GelMa cylinders were observed by field emission scanning electron microscopy (HITACHI SUS8010, Japan).

Swelling experiment and degeneration experiment: The quality of GelMa hydrogel after freeze-drying or immersed in PBS for a specific time was measured. The water on the surface of GelMa before measurement was wiped off to ensure the integrity of the hydrogel structure.

Release experiment: The freeze-dried GelMa hydrogel loaded with PDGF-BB in PBS was soaked. The supernatant was taken after a fixed amount of time, and the PDGF-BB Elisa Kit (MultiSciences Biotech, Hangzhou, Zhejiang, China) was used to determine the amount of cytokines released in the supernatant.

ECM scaffold was purchased from Tiantian Garden Ecological Breeding (Shaoxing, Zhejiang, China). Surface morphology of ECM scaffolds was observed by field emission scanning electron microscopy (HITACHI SUS8010, Japan).

### hBMSC culture

HBMSCs were purchased from Cyagen (Suzhou, Jiangsu, China) at the second passage and were used in the cytocompatibility and osteogenic characterization in the experiment. The cells were raised by alpha modification essential medium (ɑ-MEM) containing 10% fetal bovine serum (Gibco, USA), 1% penicillin/streptomycin (Invitrogen, USA) and 1% penicillin/streptomycin (Invitrogen, the USA). The basic culture medium was replaced 3 times a week. The third passage of hBMSCs was used in all of the vivo experiments.

Osteogenic differentiation culture: hBMSCs within the 5th generation were implanted into the corresponding well plates according to experimental groups. After cell adhesion and growth, osteogenic differentiation was induced by osteogenic differentiation medium. The osteogenic differentiation medium was consists of the following parts: MSC basic medium, 2 mM L-glutamine (L-glu), 100 IU/ml penicillin, 100 μg/ml streptomycin, 0.1 μM dexamethasone, 10 mM β-glycerol phosphate, and 50 μM ascorbic acid 2-phosphate. Then, 0.22-μm filters were used to filter sterilize before storing frozen aliquots in a −20 °C freezer. In the process of osteogenic differentiation culture, the medium was changed once every 2 d until the sample was collected at the experimental time node.

### Cell viability assay

PDGF-BB was purchased from R&D Systems (Shanghai, China), and PDGFR-β inhibitor, also called SU-16f, was purchased from MedChemExpress (USA).

Cell counting kit-8 assay: Cell counting kit-8 reagent from Biosharp (Shanghai, China) was used by instructions to evaluate cytotoxicity of different groups (control group, PDGF-BB group, and PDGFR-β inhibitor group) in different mediums (basic medium and osteogenic medium) respective at time points of 1, 3, 5 and 7 d. At each time point of the day, the supernate of the culture medium was collected and measured at 450 nm by a spectrophotometric microplate reader (Bio-Rad 680, USA) after 2- to 4-h incubation.

Life & Dead assay: Calcein/PI cell viability and cytotoxicity assay kit was purchased from Beyotime (Shanghai, China). Plant hBMSCs in the lower room of the Transwell plate with the density of 2.0 × 10^4^ cells/ml, and the upper Transwell chamber was placed with a piece of GelMa hydrogel of each group (blank group, GelMa group, and GelMa/PDGF-BB group). After washing the medium, we added an appropriate volume of freshly prepared Calcein-AM/PI detection working solution and incubated it at 37 °C for 30 min in the dark. Then, we used an inverted fluorescence microscope (Leica Inc., Germany) to detect cells after incubation (Calcein-AM with green fluorescence, Ex/Em = 494/517 nm; PI with red fluorescence, Ex/Em = 535/617 nm), and live cells showed green while dead cells showed red.

### Cell proliferation assay

EdU assay: EdU cell proliferation kit was purchased from Beyotime (Shanghai, China). Plant hBMSCs in proper plate with the density of 2.0 × 10^4^ cells/ml under the experimental design (control group, PDGF-BB group, and PDGFR-β inhibitor group). In accordance with the instructions in the kit, freshly prepared detection reagents were added one by one and the cell nuclei was finally stained with 4′,6-diamidino-2-phenylindole (DAPI). Then an inverted fluorescence microscope (Leica Inc., Germany) was used. DAPI was blue fluorescence with a maximum excitation wavelength of 346 nm and a maximum emission wavelength of 460 nm, the active component of cell proliferation called Azide-488 was between the maximum excitation wavelength of 495 nm, and the maximum emission wavelength is 519 nm.

### Osteogenic activity assay

ALP staining and quantitative analysis: The ALP staining assay was performed in examination of osteogenesis activity of different groups. The cells in the medium of a specific group were cultured for 7 d and stained according to the instructions of the ALP staining kit (Beyotime, Shanghai, China) after incubation of 1% Triton X-100 for 10 min (min). Quantitative assay was performed using alkaline phosphatase detection kit (Beyotime, Shanghai, China). Para-nitrophenol(*p*-nitrophenol) can be generated from para-nitrophenyl phosphate under alkaline phosphatase for determination of absorbance at 400 to 415 nm.

ARS and quantitative analysis: The alizarin red was performed in the examination of the differentiation of hBMSCs by aggregating with calcium to form chelates. The same cultured cells like ALP staining in the proper plate with medium according to specific groups for 14 d. Then, the experimental cells were rinsed by ultrapure water 3 times and then incubated with 4% paraformaldehyde for 15 min. After fixing and washing, the cells were incubated with alizarin red for 30 min and washed with sterile water. For quantitative analysis, alizarin red was dissolved with 10% cetylpyridinium chloride, and then the lysate was detected at 562 nm with the microplate reader.

### Ribonucleic Acid (RNA) acquirement and quantitative real-time PCR (qRT-qPCR) assay of osteogenesis-related genes

hBMSCs (500 μl) with a concentration of 10^4^ cells cm^−2^ were cultured on different samples for 3 and 5 d, respectively. The culture medium was removed and then rinsed with PBS 3 times. Total RNA was extracted by TRIzol reagent with an RNA extract kit (Bioteck Co. Germany). Then, RNA-reversed transcription was performed with Prime-ScriptTMRT reagent kit (Takara Co. Beijing, China). The condition of 2-step cycling amplification was set as 95 °C for 30 s, following by 45 cycles of 95 °C for 5 s and 60 °C for 30 s. The Bio-Rad CFX Manager system was applied to perform qRT-PCR. Additionally, Glyceraldehyde-3-phosphate dehydrogenase (GAPDH) was selected as the reference gene and the sequences of different primers were displayed in the below Table.

**Table.** Primer sequences for RT-qPCR

Primer sequence name	Primer sequence (5′ to3′)
ALP (F)	ACCACCACGAGAGTGAACCA
ALP (R)	CGTTGTCTGAGTACCAGTCCC
Col-1 (F)	ACAGGCATAAAAGGCCCACT
Col-1 (R)	GGACTTCCGTAGCCTGGTTT
Sp-7 (F)	CCTGCGACTGCCCTAATT
Sp-7 (R)	GCGAAGCCTTGCCATACA
Runx-2 (F)	ACTTCCTGTGCTCGGTGCT
Runx-2 (R)	GACGGTTATGGTCAAGGTGAA
GAPDH (F)	GAAAGCCTGCCGGTGACTAA
GAPDH (R)	TGGAATTTGCCATGGGTGGA

### Protein extraction and WB assay of signaling pathway

Isolated total protein of hBMSCs from each group by Radio Immunoprecipitation Assay (RIPA) buffer with a concentration of 1 mM phenylmethanesulfonylfluoride fluoride and measured protein concentration by Bicinchoninic Acid (BCA) protein assay kit from Beyotime (Shanghai, China). Then, separated proteins by sodium dodecyl sulfate-polyacrylamide gel electrophoresis and transferred to a polyvinylidene difluoride membranes (Bio-Rad, USA). After blocking with 5% nonfat milk, the membranes were incubated with primary antibodies as GAPDH (1:1,000), extracellular regulated protein kinases 1/2 (ERK 1/2) (1:1,000) (Cell Signaling Technology/CST, USA), and Smad1/5/8 (1:1,000) (Abcam, the UK) at 4 °C overnight. The respective secondary antibodies were applied after incubation. The selected bands were cut and then detected with electrochemiluminescence plus reagent (Invitrogen, the USA).

### Balb/c mouse skull CSD model of bone defect

All animal procedures were approved and guided by Zhejiang University Ethics Committee of experimental animals. All animals in this research received humanitarian care. Adult male Balb/C mice (20g±2g) were purchased from Vital River (Beijing, China) and raised in the Animal Experiment Center of the Second Affiliated Hospital of Zhejiang University (SAHZJU). The mice were housed in a controlled environment under standard conditions of temperature and humidity and an alternating 12-h light and dark cycle with supply water and food. The animal mice were randomly divided into groups before bone defect operation. After mice were anesthetized with 1% sodium pentobarbital by intraperitoneal injection, the cranial parietal hairs were gently removed with a dermatome. A cotton ball was dipped in iodophor solution to sterilize the cranial top surgical area, and then lidocaine was injected locally for local anesthesia. Then, the calvaria was exposed layer by layer, and a 3.5-mm-diameter dental trephine was used to create the bone defect area in the middle of the calvaria without damaging the mouse brain tissue. After the materials were placed in groups according to the groups, the surgical area was sutured layer by layer and ear tags were applied after secondary disinfection.

### Micro-CT analysis and quantitative analysis

After removing the skulls of each group of mice at a fixed experimental time, samples were fixed in 4% paraformaldehyde for 3 d and then converted to 75% ethanol for storage. In this experiment, the micro-CT equipment of the International Institute of Nanomaterials of Zhejiang University was used for scanning with scanning energy of 55-kV peak (kVp) and intensity of 145 ms. After scanning mouse animal specimens, each defect skull was reconstructed. As the reconstructions completed, the image was rotated to align the longitudinal axis of the bone with the Y-axis of the image and a cylindrical mask was artificially placed on the defect to fit the bone damage area of ​​the critical bone defect. Then, the micro-CT supporting analysis software Imalytics Preclinical was used to perform various data analysis, including bone mineral density, bone volume, bone surface, tissue volume, trabecular thickness, and trabecular separation.

### Histology and immunohistochemistry

The mouse skull specimens were decalcified with 10% ethylene diamine tetraacetic acid (EDTA) decalcification solution. After the decalcification was completed, the specimens were cut into appropriate shapes and then placed in embedding clips for dehydration treatment. The specimens were embedded in liquid paraffin at an appropriate prevention and treatment position. Sections of embedded mouse calvarial blocks were made, each slice 4.5 μm thick.

Histology: Standard single deparaffinization of prepared paraffin sections and then stained each sample with H&E and Masson staining. The inflammatory cell infiltration and cellularity and morphology of skull were examined in a blinded manner using a microscope (Leica Inc., Germany).

Immunofluorescence assay: Standard single deparaffinization of prepared paraffin sections. Antigen retrieval was performed with trypsin without EDTA after covering the specimen with hydrogen peroxide. The membrane was covered with 01% Triton and then washed 3 times with PBS. After blocking with fetal sheep serum at 37 °C, the primary antibody was directly covered with the concentration of CD31 (1:200), Erk 1/2 (1:200), Smad1/5/8 (1:200), Col1a1 (1:200), Runx-2 (1:200), and Osterix (1:200) (Abcam, the UK) at 4 °C overnight. After washed 3 times in PBS, the membrane was covered with the corresponding fluorescent secondary antibody and incubated at 37 °C for 2 h and then dipped 3 times again. Slides with anti-fluorescence-destructed mounting medium containing Dapi were mounted. Then, an inverted fluorescence microscope was used (Leica Inc., Germany).

### Statistical analysis

All data were evaluated by 1-way analysis of variance (ANOVA) followed by post hoc Tukey’s multiple comparison test at least 3 experiments of similar results performed in triplicate unless otherwise indicated. All data were presented as mean ± standard deviation (independent samples, *n* ≥ 3) with a difference of *P < 0.05 which was considered significant.

## Data Availability

The data that support the findings of this study are available from the corresponding author upon reasonable request.
