# Supplementary material for: PDGFR in PDGF-BB/PDGFR Signaling Pathway Does Orchestrates Osteogenesis in a Temporal Manner
Source: Research (Wash D C). 2023 May 5;6:0086. doi: 10.34133/research.0086 (PMC10202377; doi:10.34133/research.0086)
Supplement: Supplementary Materials — Figs. S1 to S10. [file research.0086.f1.docx]

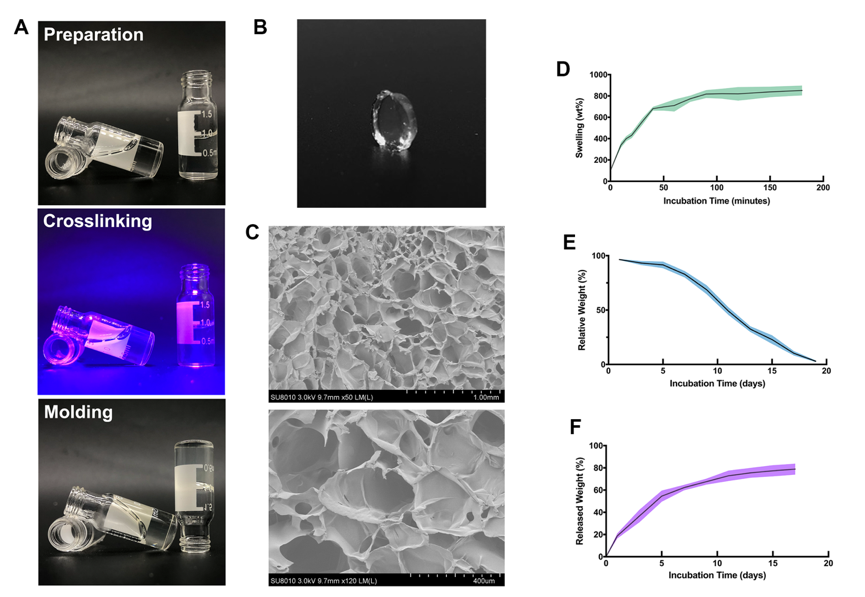


Supplemental Figure 1. Characterization of GelMa hydrogel equipped with PDGF-BB.

(A) The preparation process of the GelMa hydrogel (successively including three stages of Preparation, Crosslinking and Molding).

(B) The gross image of GelMa cylinder.

(C) The SEM of GelMa with respective 50x and 120x image.

(D) The swelling ratio of GelMa.

(E) The degradation profile of GelMa.

(F) The release curves of PDGF-BB releasing from the GelMa cylinder.


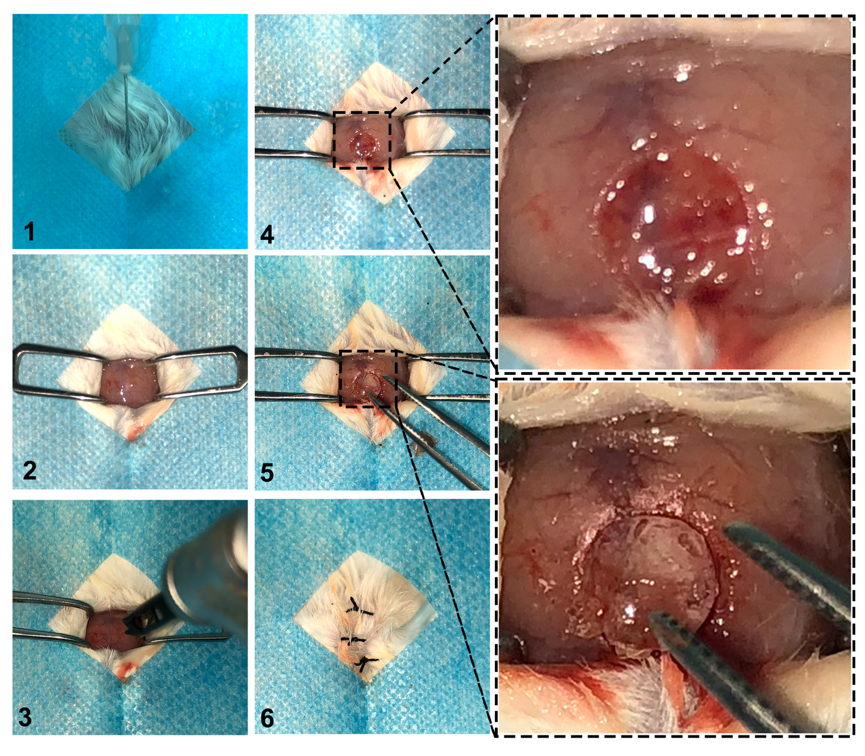


Supplemental Figure 2. The process of mouse calvarial CSD model operation with calvarial magnification image of critical bone sclerite stripped and GelMa inserted.


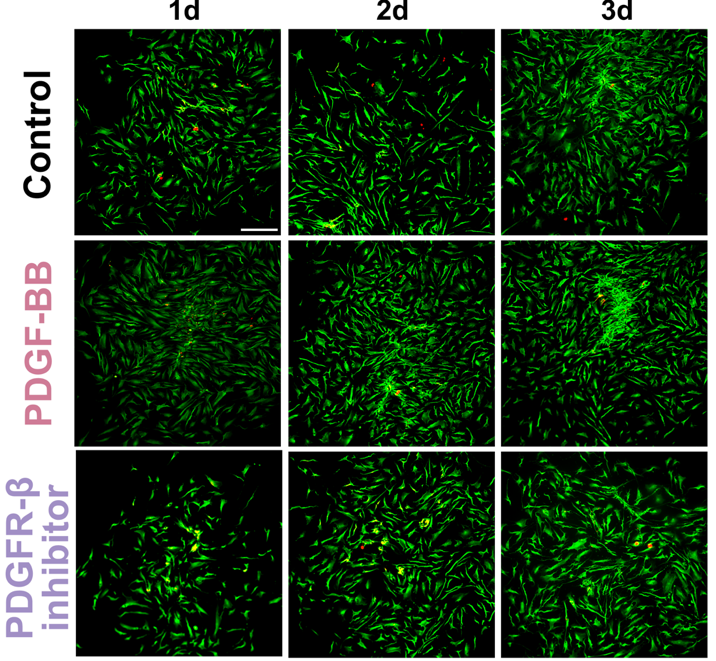


Supplemental Figure 3. Live and dead assay of Control, PDGF-BB (10 ng/ml) and PDGFR-β inhibitor (SU-16f)(5 μM) each in 1, 2 and 3 days. Scale bar 40 μm.


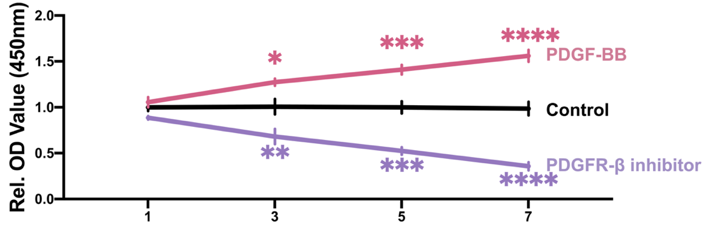


Supplemental Figure 4. The CCK-8 assay of Control, PDGF-BB (10 ng/ml) and PDGFR-β inhibitor (SU-16f) (5 μM) each in 1, 3, 5 and 7 days. Data are means ± SEM. One-way ANOVA. ns P≥0.05; *P<0.05; **P < 0.01; ***P < 0.001.


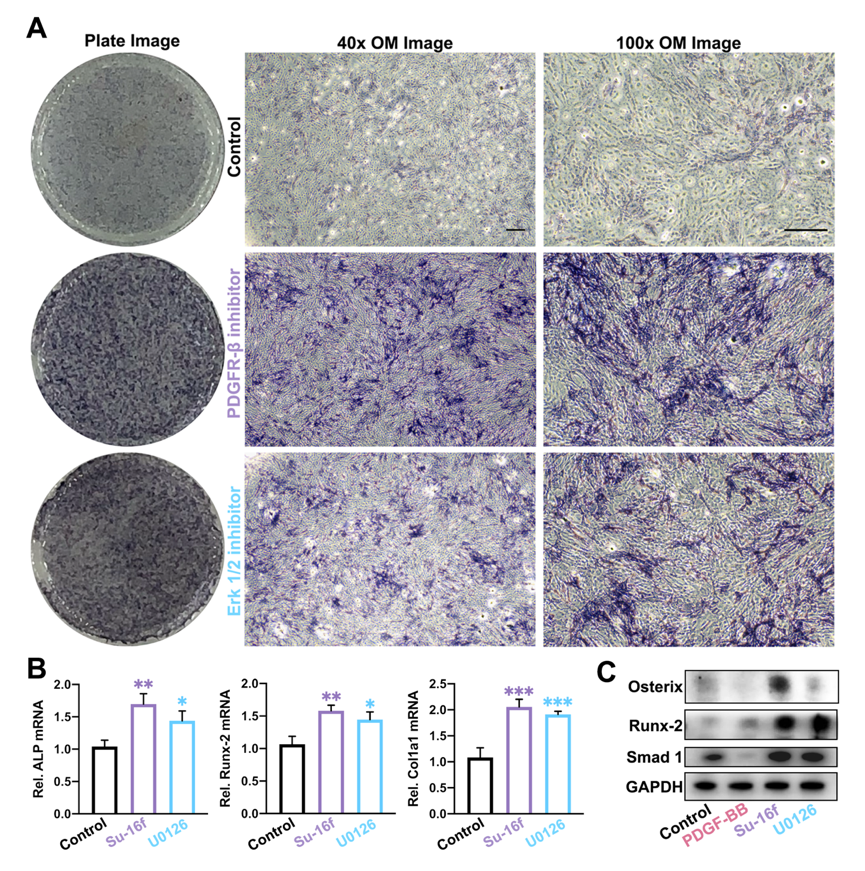


Supplemental Figure 5. The Erk 1/2 inhibitor (U0126) has a similar effect as the PDGFR-beta inhibitor (SU-16f) in promoting osteogenic characterization of hBMSCs.

(A) ALP staining of the groups (Control, black; 5 μM PDGFR-β inhibitor, purple; 10 μM Erk 1/2 inhibitor, blue) with low (40x) and high (100x) magnification of optical micrograph. Scale bar 50 μm.

(B) PCR analysis of ALP, Col1a1 and Runx-2 mRNA of the groups with 5 days. Data are means ± SEM. One-way ANOVA. ns P≥0.05; *P<0.05; **P < 0.01; ***P < 0.001.

(C) Western blot band of Smad 1, Runx-2 and Osterix of groups (Control, black; 10 ng/ml PDGF-BB, red; 5 μM PDGFR-β inhibitor, purple; 10 μM Erk 1/2 inhibitor, blue) with 7 days.


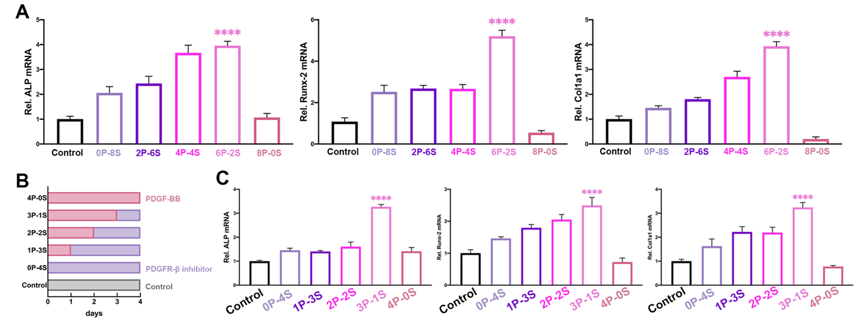


Supplemental figure 6. PCR analysis of ALP, Col1a1 and Runx-2 mRNA of the groups under temporal treatment.

(A) PCR analysis of ALP, Runx-2 and Col1a1 mRNA of the groups of 8 days. (Control, black; 0P-8S, lilac; 2P-6S, modena; 4P-4S, amaranth; 6P-2S, light red; 8P-0S, red). Data are means ± SEM. One-way ANOVA. ns P≥0.05; *P<0.05; **P < 0.01; ***P < 0.001.

(B) The schematic diagram of temporal control grouping with total time of 4 days (number before P or S respectively means cultured days of 10 ng/ml PDGF-BB or 5 μM SU-16f in the medium).

(C) PCR analysis of ALP, Runx-2 and Col1a1 mRNA of the groups of 4 days. (Control, black; 0P-4S, lilac; 1P-3S, modena; 2P-2S, amaranth; 3P-1S, light red; 4P-0S, red). Data are means ± SEM. One-way ANOVA. ns P≥0.05; *P<0.05; **P < 0.01; ***P < 0.001.


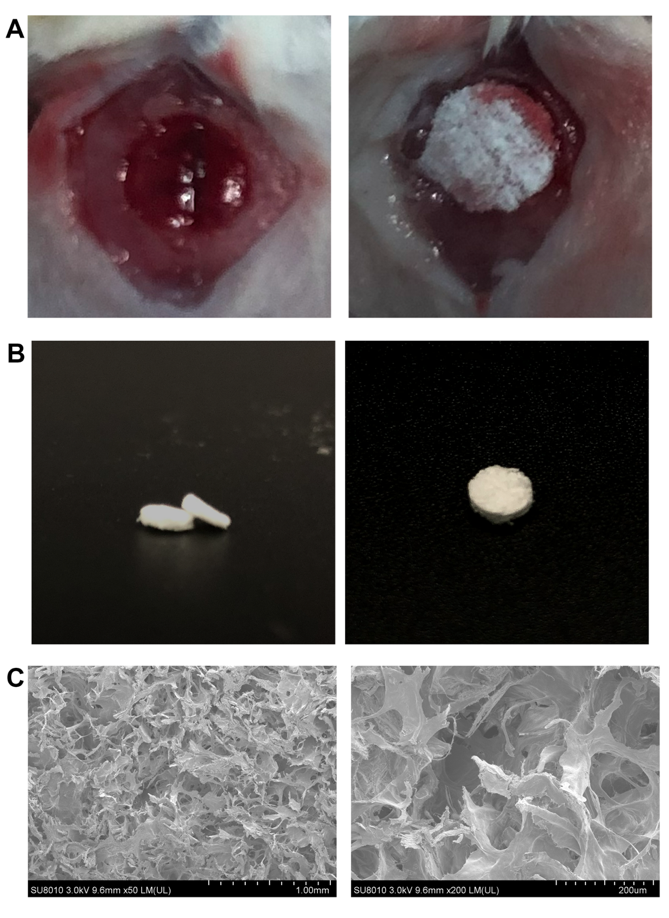


Supplemental figure 7. Characterization of scaffold materials including implantation in animals, gross photographs and SEM characterization.

(A) Illustration of the critical bone defect of the mouse skull implanted with ECM scaffolds.

(B) The gross image of ECM cylinder.

(C) The SEM of GelMa with respective 50x and 200x image.


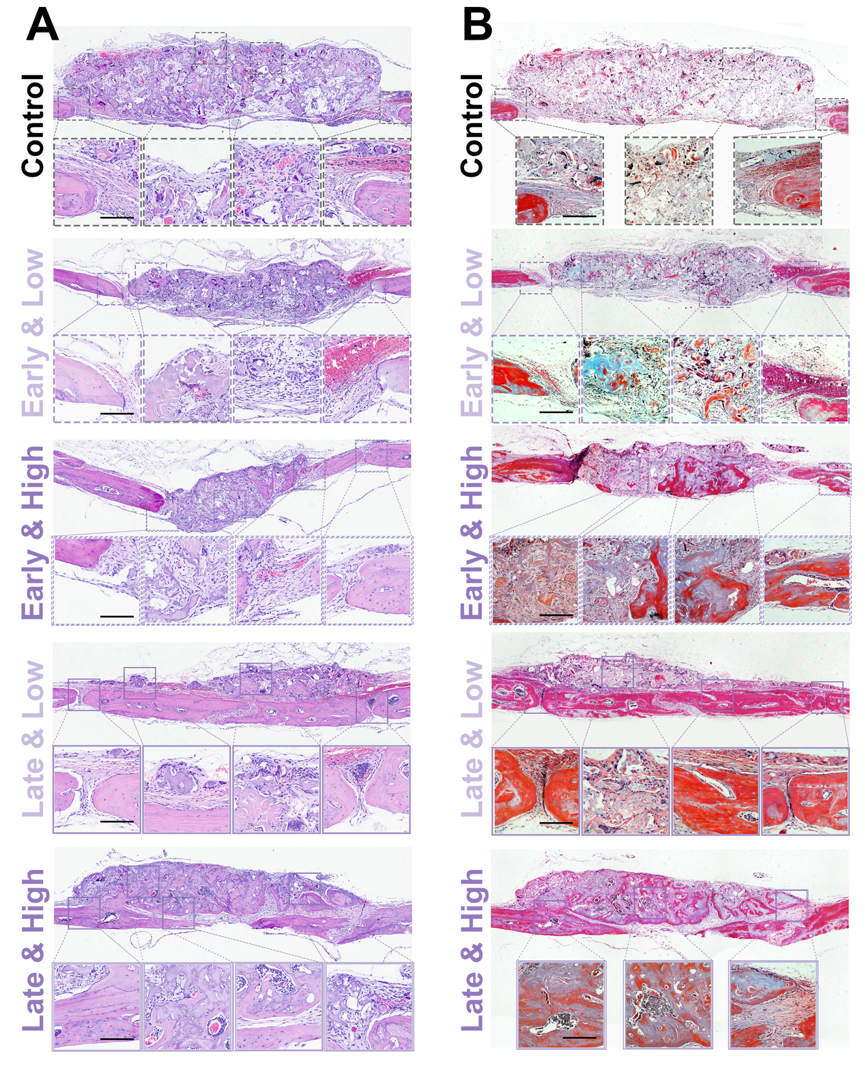


Supplemental Figure 8. Detailed data of pathology staining of Figure 4D and Figure 4E under the groups shown in Figure 4.

(A) The HE staining image of each group (Control, black dotted line; Early/Low, purple single dotted line; Early/High, purple double dotted line; Late/Low, purple single full line; Late/High, purple double full line). Scale bar 50 μm.

(B) The Masson staining of each group with red line (Control, black dotted line; Early/Low, purple single dotted line; Early/High, purple double dotted line; Late/Low, purple single full line; Late/High, purple double full line). Scale bar 50 μm.


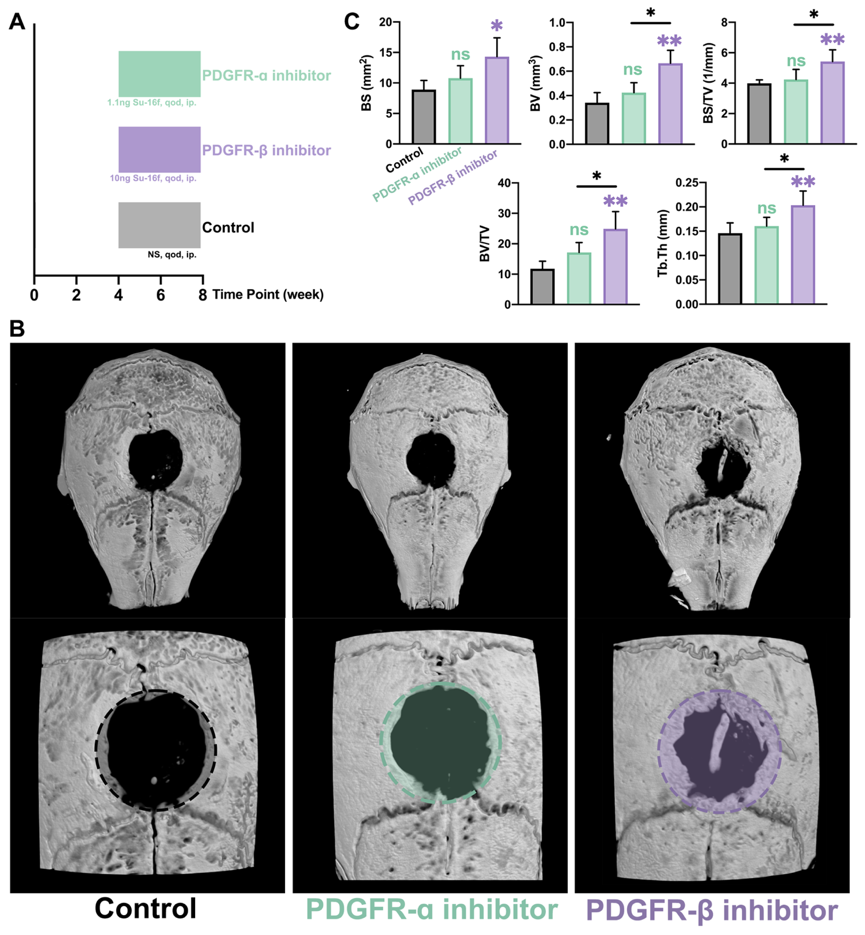


Supplemental Figure 9. In the same late stage of bone defect, PDGFR-β inhibitor (SU-16f) can promote critical bone defect healing in mice, but PDGFR-ɑ inhibitor (AP24534) can not.

(A) The drug delivery schematic diagram of Control, PDGFR-ɑ inhibitor (1.0 ng AP24534, qod., ip.) and PDGFR-β inhibitor (10 ng SU-16f, qod., ip.).

(B) The reconstructions of representative calvarial models (average of the individual samples) measured by Micro-CT. Original defect area is shaded with a dashed outline (Control, black; PDGFR-ɑ inhibitor, green; PDGFR-β inhibitor, purple).

(C) The quantitative analysis of Micro-CT reconstruction of CSD model. Data are means ± SEM. n = 5 or 6. One-way ANOVA. ns P≥0.05; **P < 0.01.


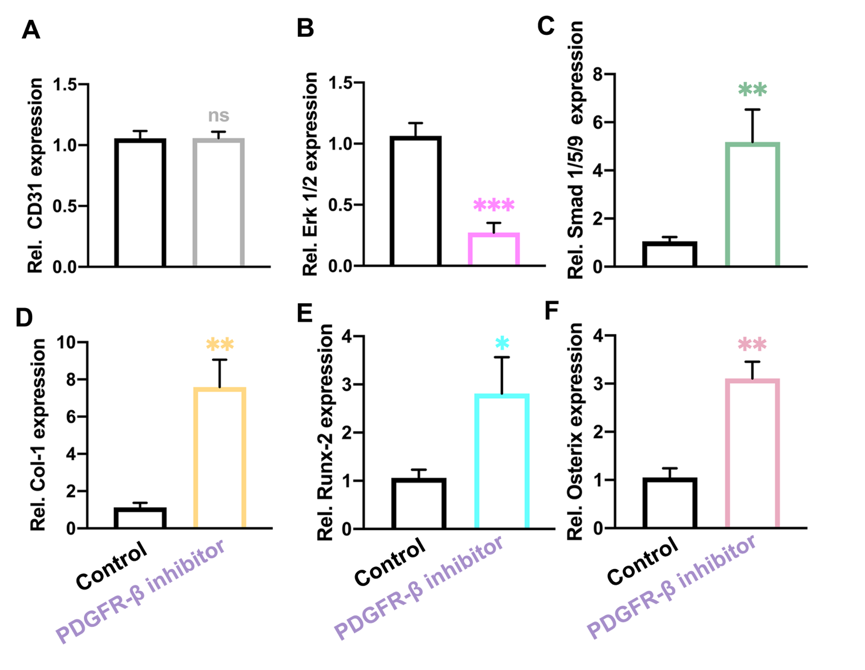


Supplemental Figure 10. The quantitative analysis of different markers of immunofluorescence staining shown in Figure 6.

(A) The quantitative analysis of CD31 maker of immunofluorescence staining between Control group (black) and PDGFR-β inhibitor group (white). Data are means ± SEM. n = 3. Student’s T-test. ns P≥0.05; *P<0.05; **P < 0.01; ***P < 0.001.

(B) The quantitative analysis of Erk 1/2 maker of immunofluorescence staining between Control group (black) and PDGFR-β inhibitor group (amaranth). Data are means ± SEM. n = 3. Student’s T-test. ns P≥0.05; *P<0.05; **P < 0.01; ***P < 0.001.

(C) The quantitative analysis of Smad 1/5/9 maker of immunofluorescence staining between Control group (black) and PDGFR-β inhibitor group (green). Data are means ± SEM. n = 3. Student’s T-test. ns P≥0.05; *P<0.05; **P < 0.01; ***P < 0.001.

(D) The quantitative analysis of Col-1 maker of immunofluorescence staining between Control group (black) and PDGFR-β inhibitor group (yellow). Data are means ± SEM. n = 3. Student’s T-test. ns P≥0.05; *P<0.05; **P < 0.01; ***P < 0.001.

(E) The quantitative analysis of Runx-2 maker of immunofluorescence staining between Control group (black) and PDGFR-β inhibitor group (cyan). Data are means ± SEM. n = 3. Student’s T-test. ns P≥0.05; *P<0.05; **P < 0.01; ***P < 0.001.

(F) The quantitative analysis of Osterix maker of immunofluorescence staining between Control group (black) and PDGFR-β inhibitor group (red). Data are means ± SEM. n = 3. Student’s T-test. ns P≥0.05; *P<0.05; **P < 0.01; ***P < 0.001.
